# Propensity Score-Matched Analysis of Lesion Patterns in Stroke Patients With Patent Foramen Ovale and Patients With Spontaneous Intracranial Artery Dissection

**DOI:** 10.3389/fneur.2019.00418

**Published:** 2019-04-24

**Authors:** Yangyang Huang, Yifan Cheng, Bei Shao, Xuanyou Zhou, Huazheng Liang, Jianhua Zhuang, Yong Bi

**Affiliations:** ^1^Department of Neurology, Shanghai Fourth People's Hospital, Tongji University, Shanghai, China; ^2^Department of Neurology, The First Affiliated Hospital, Wenzhou Medical University, Wenzhou, China; ^3^The First Clinical Medical Institute, The First Affiliated Hospital, Wenzhou Medical University, Wenzhou, China; ^4^Department of Neurology, Changzheng Hospital, Second Military Medical University, Shanghai, China

**Keywords:** patent foramen ovale, spontaneous intracranial artery dissection, ischemic stroke, embolism, DWI

## Abstract

**Aims:** To investigate the relationship between clinical and imaging features of stroke patients with patent foramen ovale (PFO) and those with spontaneous intracranial artery dissection (SIAD).

**Materials and methods:** We retrospectively examined both clinical and imaging results of 40 stroke patients with PFO and 29 with SIAD. To reduce selection bias, we conducted a propensity score-matching analysis. The patients' propensity scores were estimated using a logistic regression model based on the following variables: age, sex, hypertension, diabetes mellitus, hypercholesterolemia, cigarette smoking, stroke histories, and their NIHSS scores. We compared the pattern of cerebral DWI lesions between patients with PFO and those with SIAD.

**Results:** After propensity score matching, 21 pairs of patients were selected. Clinical characteristics of the 2 groups were well matched. The distribution of DWI lesion patterns differed between the 2 groups. Single lesions (cortical or subcortical) were more frequently observed in the PFO group than in the SIAD group (*P* = 0.026). Multiple lesions in one vascular territory occurred more frequently in the SIAD group than in the PFO group (*P* = 0.035).

**Conclusion:** The present study suggests that lesion patterns observed from DWI of patients with PFO and SIAD might provide clues to the etiology of infarcts. Single lesions (cortical or subcortical) might be a typical feature of PFO associated strokes, while multiple lesions in one vascular territory might be a specific feature of SIAD associated strokes.

## Introduction

Both patent foramen ovale (PFO) and spontaneous intracranial artery dissection (SIAD) are important stroke risk factors, especially in young and middle-aged adults (1–3). About 25% of patients with ischemic stroke are cryptogenic (4), and PFO is present in ~54% of patients with cryptogenic stroke under 54 yeas (5). PFO and SIAD might result in strokes through different mechanisms. Promptly identifying the difference in the underlying mechanisms among young and middle-aged stroke patients will facilitate selection of the best therapy for the primary disease and prevention of recurrent strokes.

A prior study has assessed brain Magnetic resonance imaging (MRI) features of stroke patients due to PFO and SIAD and found that, a single non-territorial infarct seemed to favor strokes due to PFO, whereas territorial infarcts (with or without additional smaller lesions in the same territory) were more likely to occur in patients with arterial dissection (6). However, this study did not individually compare between patients with carotid dissection, vertebral dissection, and those with PFO, respectively, though the former two types of patients had different mechanisms of stroke pathogenesis. In SIAD, spontaneous cervical artery dissection showed different clinical and imaging features from spontaneous vertebral artery dissection (7, 8). Artery-to-artery embolism and reduced blood flow due to the primary lesion are the two basic mechanisms with which spontaneous cervical artery dissection led to strokes (9, 10), whereas artery-to-artery embolism is considered to be the primary mechanism with which spontaneous vertebral artery dissection led to strokes (11, 12). Therefore, it might be more reliable to individually analyze the imaging characters of carotid dissection and vertebral dissection patients. The other thing to note is that the baseline data of some studies are not consistent with each other, which leads to systemic bias in data analysis and subsequently the findings from these studies are not trustworthy.

In the present study, we aimed to analyze the difference in imaging findings between stroke patients with PFO and those with carotid dissection or vertebral dissection through propensity analysis. Results from this study will facilitate the diagnosis of these diseases in stroke patients and initiate appropriate therapies.

## Materials and Methods

### Subjects

Stroke patients with PFO and SIAD, admitted to the Department of Neurology and Neurosurgery, the First Affiliated Hospital of Wenzhou Medical University between August 2010 and June 2018, were included in the present retrospective study. This study was approved by the Human Ethics Committee of the First Affiliated Hospital of Wenzhou Medical University.

Inclusion criteria for stroke patients due to SIAD were: (1) definite angiographic findings of dissection in intracranial arteries; (2) presence of ischemic stroke lesions on Diffusion-weighted imaging (DWI); (3) according to the TOAST criteria, no definite causes had been identified apart from dissection after a standardized workup; (4) PFO was excluded by transesophageal echocardiography (TEE) and transcranial Doppler (TCD); (5) Spontaneous cervical artery dissection was diagnosed based on ≥1 of the following 3 criteria: (1) intimal flap visible on carotid ultrasound; (2) mural hematoma visible on MRI or CT; (3) a non-atherosclerotic, tapered, flame-shaped ICA-occlusion or a string-like ICA-stenosis (7). A total of 31 patients were diagnosed with SIAD, one patient was excluded due to absence of lesions on DWI, and another patient excluded due to an extracranial artery dissection. As a result, 29 patients were included in the “SIAD group.”

**Figure 1:**
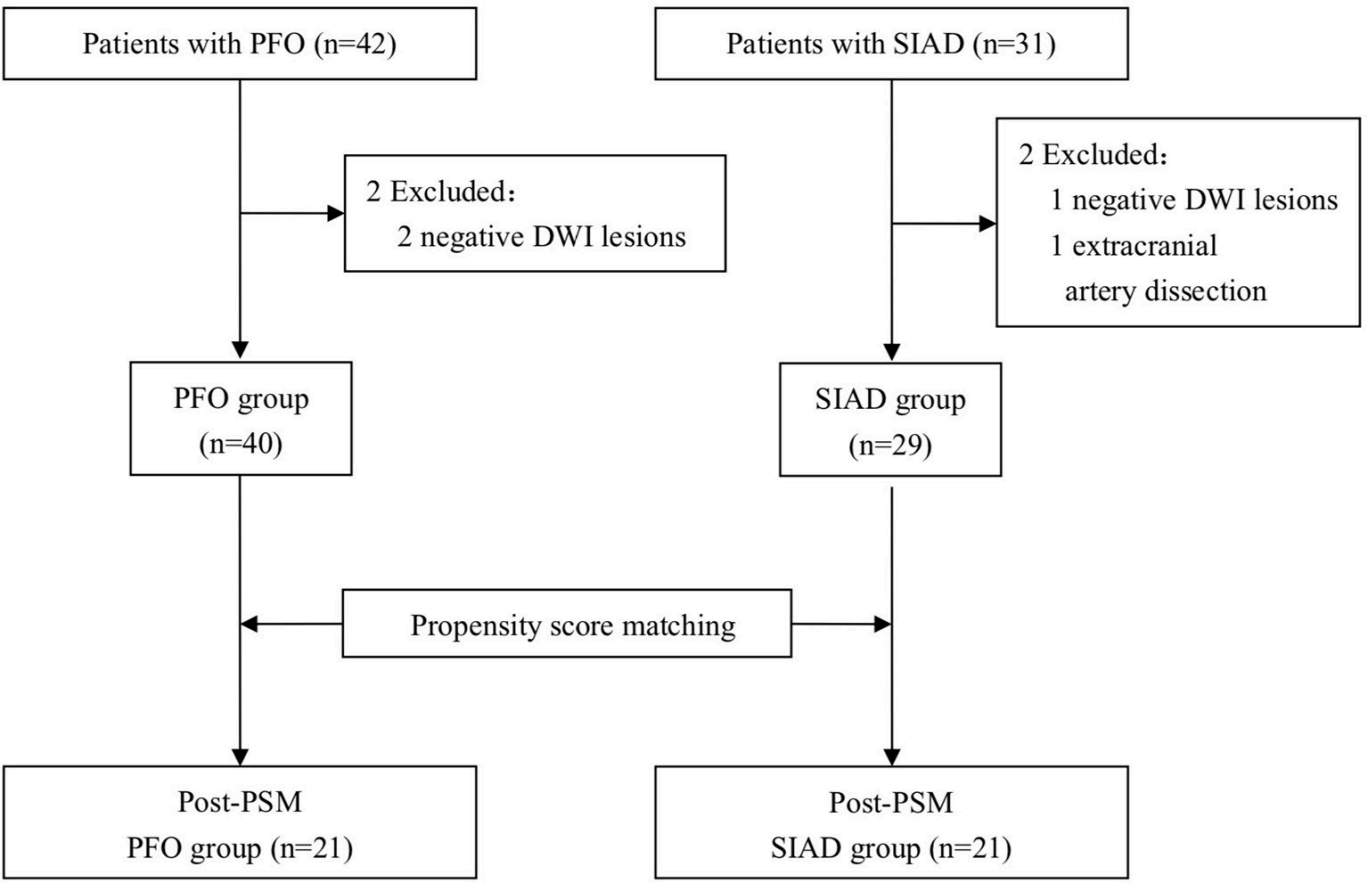
Flowchart of the patient selection process.

Inclusion criteria for stroke patients with PFO were: (1) presence of ischemic stroke lesions on DWI; (2) according to the TOAST criteria, no definite causes had been identified apart from PFO after a standardized etiological workup; (3) evaluated with a standard stroke protocol including DWI sequence; (4) The presence of PFO was confirmed by transesophageal echocardiography (TEE) and the presence of right-to-left shunt was tested by transcranial Doppler (TCD) (13, 14). Forty two patients with PFO and no other definite stroke causes were chosen, but 2 were excluded due to absence of lesions on DWI, the remaining 40 patients were included in the “PFO group.” A flowchart of the selection process was shown in Figure 1.

We reviewed age, sex, medical histories, stroke histories, physical examination, laboratory test results, and stroke severity measured by the National Institute of Health Stroke Scale (NIHSS) score. Conventional vascular risk factors were identified and listed below: hypertension, diabetes mellitus, hypercholesterolemia, cigarette smoking.

All patients underwent brain Magnetic resonance imaging, transcranial doppler, carotid and vertebral artery ultrasound, magnetic resonance angiography/computer tomography angiography or digital subtraction angiography to exclude infarction due to extracranial or intracranial atherosclerosis. Chest x-ray, electrocardiogram, transthoracic echocardiography, and transesophageal echocardiography, 24 h electrocardiogram were applied to exclude infarction due to emboli from the heart. Laboratory tests for hypercoagulation states, vasculitis, or metabolic disorders were requested at the discretion of the treating physician.

### Brain MR Imaging

MRI was performed using a 3.0-T MRI unit (Signa HDx; GE Medical Systems, Milwaukee, WI). Data were obtained from the DWI sequence. DWI was performed with a repetition time of 5,000 ms, echo time of 84 ms, field of view of 24 × 24 mm, matrix number of 128 × 128, and slice thickness of 5 mm.

**Figure 2:**
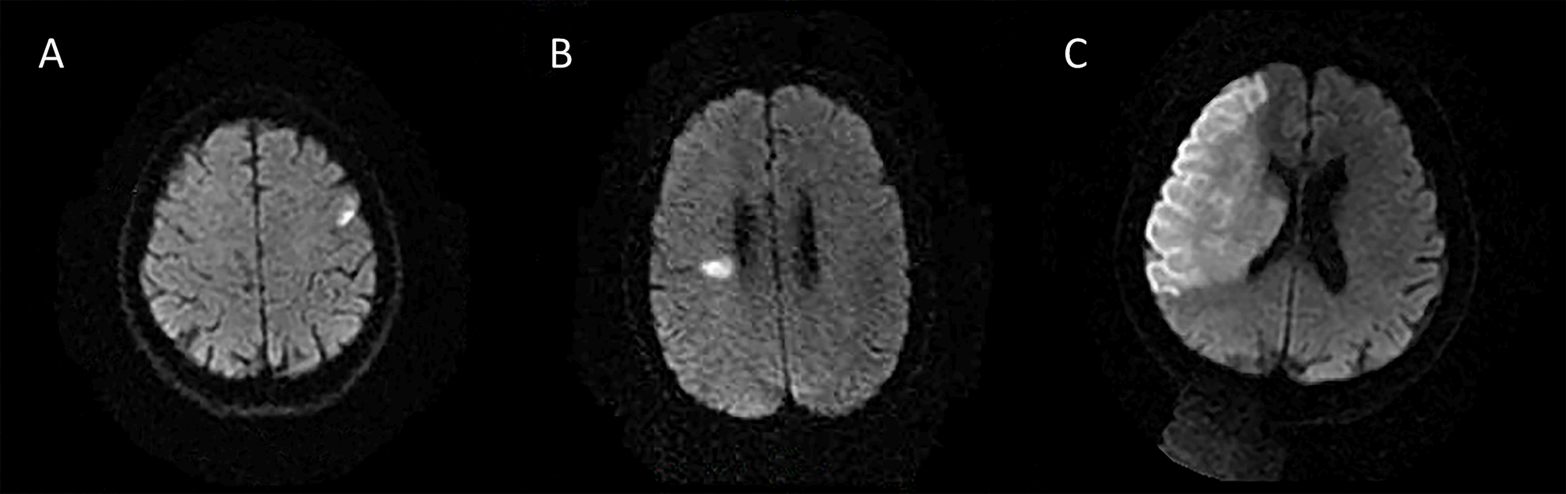
Single lesions **(A)** (Pattern 1): single lesions (cortical). **(B)** (Pattern 1): single lesions (subcortical). **(C)** (Pattern 2): single lesions (corticosubcortical).

**Figure 3:**
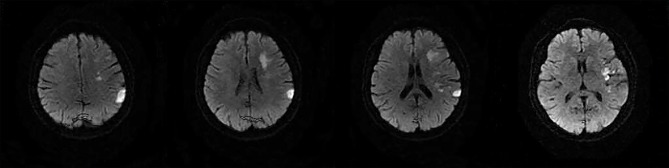
(Pattern 3): multiple lesions in one vascular territory.

**Figure 4:**
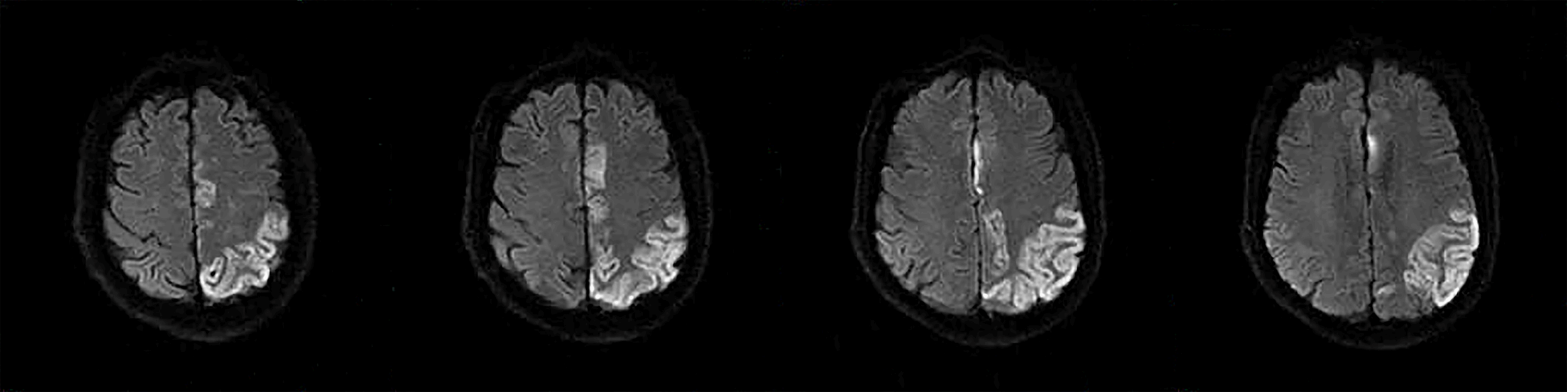
(Pattern 4): multiple lesions in multiple vascular territories (same side).

**Figure 5:**
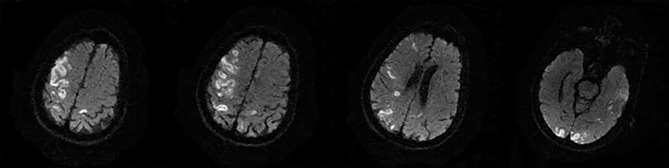
(Pattern 5): multiple lesions in multiple vascular territories (different sides).

The following 5 lesion patterns on DWI were shown in Figures 2–5, using a published classification system: (1) Pattern 1: single lesions (cortical, or subcortical); (2) Pattern 2: single lesions (corticosubcortical); (3) Pattern 3: multiple lesions in one vascular territory (anterior or posterior circulation); (4) Pattern 4: multiple lesions in multiple vascular territories (same side); (5) Pattern 5:multiple lesions in multiple vascular territories (different sides) (15, 16). Multiple lesions were defined as 2 or more isolated DWI-positive lesions within the same or multiple vascular territories (9). Multiple lesions were classified into unilateral anterior circulation, posterior circulation, bilateral anterior circulations, or anterior and posterior circulations. Based on the difference in supplying arteries, vascular territories were also divided into anterior circulation (carotid artery), posterior circulation (vertebral-basal artery), anterior and posterior circulations (carotid and vertebral-basal arteries).

### Propensity Score Matching

We performed Propensity score matching (PSM) to reduce the possibility of selection bias and to adjust significant differences in baseline characteristics between the PFO and SIAD groups. The propensity score was estimated using a logistic regression model based on the following 8 variables: age, sex, hypertension, diabetes mellitus, hypercholesterolemia, cigarette smoking, stroke histories, and NIHSS scores. One-to-one pair matching was performed without replacement using a caliper of 0.20.

### Statistical Analysis

Statistical analyses were performed using the statistical software package SPSS version 22.0. Continuous variables were presented as mean ± SD; categorical variables were expressed as frequencies. The statistical significance for inter-group differences was tested by the **χ^2^** test for categorical variables, the ***t***-test for continuous variables, and the Mann–Whitney test for non-normal variables (e.g., NIHSS). The ROC curve was produced to predict the likelihood of right diagnosis of the underlying conditions based on findings from specific imaging results.

## Results

### Demographical Information of all Patients

Clinical characteristics of the PFO and SIAD groups were shown in Table 1. In the SIAD group, there were 17 (59%) patients with vertebral artery dissection and 12 (41%) with cervical artery dissection. The mean age of patients in the PFO group (46.85 ± 14.19 years) was significantly older than that of the SIAD group (38.79 ± 9.10 years) (*p* = 0.006). The NIHSS score of the SIAD group was significantly higher than that of the PFO group (*P* = 0.028). The percentage of males was high in both groups (73 vs. 83%) and there was no significant difference between them (*P* = 0.319). No significant difference was observed in hypertension, diabetes mellitus, hypercholesterolemia, cigarette smoking, and stroke histories between the two groups.

**Table 1:** Comparison of the clinical characteristics of patients in the two groups.

**Patient Characteristics**	**PFO, N, (%) (*n* = 40)**	**SIAD, N, (%) (*n* = 29)**	***P-*Value**
Age, y, mean ±*SD*^a^	46.85 ± 14.19	38.79 ± 9.10	0.006^*^
Male sex^b^	29 (73)	24 (83)	0.319
**Vascular risk factors^b^**			
Hypertension	15 (38)	4 (14)	0.057
Diabetes mellitus	3 (8)	2 (7)	1.000
Hypercholesterol	6 (15)	10 (34)	0.058
Cigarette smoking^b^	18 (45)	14 (48)	0.788
Stroke histories^b^	4 (10)	0	0.218
NIHSS score^c^	2.0 (1–6)	6.0 (1.5–9.5)	0.028^*^

*PFO, patent foramen ovale; SIAD, spontaneous intracranial artery dissection; NIHSS, National Institutes of Health Stroke Scale*.

* *Statistically significant (p ≤ 0.05)*.

a *Values expressed as mean ± standard deviation (SD)*.

b *Values expressed as frequencies and percentages*.

c *Values expressed as median and interquartile range*.

*P-values were determined with the use of independent **t**-test, the Pearson **χ^2^**-test (or Fisher's exact test where appropriate) and Mann–Whitney test*.

Radiological data on DWI were presented in Table 2. The distribution of DWI lesion patterns differed between the 2 groups. Single lesions (cortical or subcortical, Pattern 1) was more frequent in the PFO group than in the SIAD group (50 vs. 21%; *P* = 0.013). Multiple lesions in one vascular territory (Pattern 3) were more frequently observed in the SIAD group than in the PFO group (41 vs. 15%; *P* = 0.014). Differences in the occurrence of other lesion patterns like single lesions (corticosubcortical, Pattern 2), multiple lesions in multiple vascular territories (same side and different sides, Pattern 4 and 5) did not reach statistical significance. Additionally, there was no difference in the distribution of multiple lesions between patients with PFO and those with SIAD. Six patients with PFO had both anterior and posterior circulations involved.

**Table 2:** Comparison of the radiological data of patients in the two groups.

	**PFO, N, (%) (*n* = 40)**	**SIAD, N, (%) (*n* = 29)**	***P-*Value**
DWI lesion patterns^a^			
Single lesions (Corticosubcortical)	3 (8)	7 (24)	0.112
Single lesions (Cortical or subcortical)	20 (50)	6 (21)	0.013^*^
Multiple lesions in one vascular territory	6 (15)	12 (41)	0.014^*^
Multiple lesions in several vascular territories (same side)	2 (5)	2 (7)	1.000
Multiple lesions in several vascular territories (different side)	9 (23)	2 (7)	0.157
Distribution of multiple lesions ^a^	17 (43)	16 (55)	
Unilateral anterior circulation	8 (20)	12 (41)	0.053
Posterior circulation	7 (18)	6 (21)	0.738
Bilateral anterior circulation	4 (10)	0	0.218
Distribution of circulation^a^			
Anterior and posterior circulations	4 (10)	2 (7)	0.985
Anterior circulation	22 (55)	15 (52)	0.788
Posterior circulation	12 (30)	12 (41)	0.327
Anterior and posterior circulations	6 (15)	2 (7)	0.299

*PFO, patent foramen ovale; SIAD, spontaneous intracranial artery dissection; DWI, diffusion-weighted imaging*.

a *Values expressed as frequencies and percentages*.

*P-values were determined with the use of Pearson **χ^2^**-test (or Fisher's exact test where appropriate)*.

### Propensity Score-Matched Cohort

Patients in the PFO and SIAD groups were matched 1 to 1 using PSM to minimize the effect of confounding factors. Twenty-one pairs of patients were selected and their clinical characteristics seemed to be well matched, and no significant differences were observed between the groups after PSM (Table 3).

**Table 3:** Comparison of the clinical characteristics of patients in the two groups in the propensity score-matched cohort.

**Patient Characteristics**	**PFO, N, (%) (*n* = 21)**	**SIAD, N, (%) (*n* = 21)**	***P-*Value**
Age, y, mean ±*SD*^a^	44.38 ± 16.624	37.86 ± 9.759	0.131
Male sex^b^	16 (76)	17 (81)	1.000
**Vascular risk factors^b^**			
Hypertension	3 (14)	2 (10)	1.000
Diabetes mellitus	1 (5)	2 (10)	1.000
Hypercholesterol	5 (24)	7 (33)	0.495
Cigarette smoking^b^	9 (43)	10 (48)	0.757
Stroke histories^b^	1 (5)	0	0.311
NIHSS score^C^	2.0 (1.0–6.0)	3 (1.0–7.5)	0.713

*PFO, patent foramen ovale; SIAD, spontaneous intracranial artery dissection; NIHSS, National Institutes of Health Stroke Scale*.

a *Values expressed as mean ± standard deviation (SD)*.

b *Values expressed as frequencies and percentages*.

c *Values expressed as median and interquartile range*.

*P-values were determined with the use of independent t-test, the Pearson χ^2^-test (or Fisher's exact test where appropriate) and Mann–Whitney test*.

Post-PSM comparison of radiological data were presented in Table 4. Single lesions (cortical or subcortical, Pattern 1) were still more frequently observed in the PFO group than in the SIAD group (57 vs. 19%; *P* = 0.026). Multiple lesions in one vascular territory were more frequently observed in the SIAD group than in the PFO group (43 vs. 10%; *P* = 0.035). Unlike the full cohort, single lesions (corticosubcortical, Pattern 2) were more frequently observed in the SIAD group than in the PFO group (*P* = 0.048). Differences in the percentage of other lesion patterns like multiple lesions in multiple vascular territories (same side and different sides) did not reach statistical significance. Distribution of multiple lesions in the PSM set did not reach significant difference. Lesions in the posterior circulation territory occurred more frequently in the SIAD group than in the PFO group (57 vs. 24%; *P* = 0.028). Five patients with PFO had both anterior and posterior circulations involved.

**Table 4:** Comparison of radiological data of patients in the two groups in the propensity score-matched cohort.

	**PFO, N, (%) (*n* = 21)**	**SIAD, N, (%) (*n* = 21)**	***P-*Value**
**DWI lesion patterns^a^**			
Single lesions (Corticosubcortical)	0 (0)	5 (24)	0.048^*^
Single lesions (Cortical or subcortical)	12 (57)	4 (19)	0.026^*^
Multiple lesions in one vascular territory	2 (10)	9 (43)	0.035^*^
Multiple lesions in several vascular territories (same side)	2 (10)	1 (5)	1.000
Multiple lesions in several vascular territories (different side)	5 (24)	2 (10)	0.408
Distribution of multiple lesions ^a^	10 (48)	12 (57)	0.537
Unilateral anterior circulation	2 (10)	5 (24)	0.408
Posterior circulation	2 (10)	6 (29)	0.116
Bilateral anterior circulation	1 (5)	0	1.000
**Anterior and posterior circulations**			
Distribution of circulation^a^	5 (24)	1 (5)	0.186
Anterior circulation	11 (52)	8 (38)	0.352
Posterior circulation	5 (24)	12 (57)	0.028^*^
Anterior and posterior circulations	5 (24)	1 (5)	0.186

*PFO, patent foramen ovale; SIAD, spontaneous intracranial artery dissection; DWI, diffusion-weighted imaging*.

* *Statistically significance (p ≤ 0.05)*.

a *Values expressed as frequencies and percentages*.

*P-values were determined with the use of Pearson **χ^2^**-test (or Fisher's exact test where appropriate)*.

### Receiver Operating Characteristic (ROC) Curve

**Figure 6:**
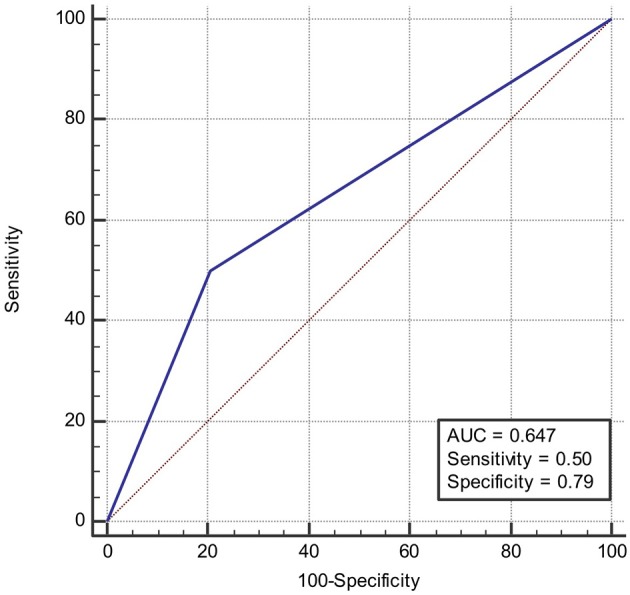
Area under the ROC curve predicts the likelihood of PFO based on patterns of single cortical or subcortical infarcts.

**Figure 7:**
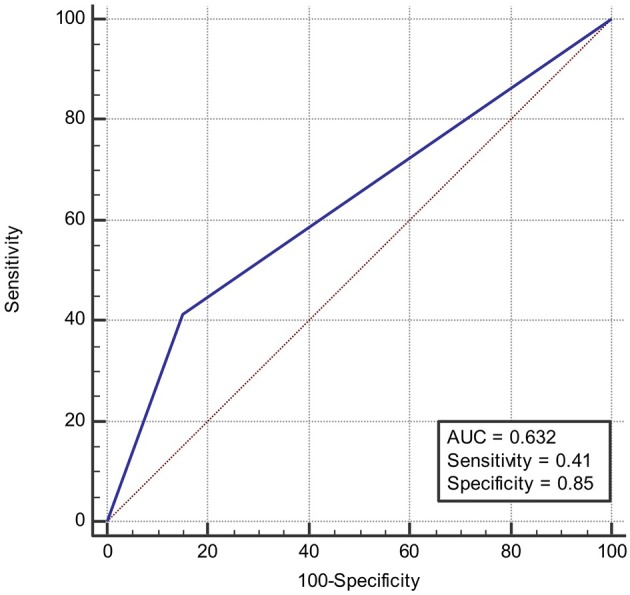
Area under the ROC curve predicts the likelihood of SIAD based on patterns of multiple infarcts in a single vascular territory.

ROC was produced to calculate the value of diagnosing PFO based on patterns of single cortical or subcortical infarcts. The area under ROC curve was 0.647 (Figure 6). Similarly, the area under ROC curve was 0.632 (Figure 7) for diagnosing SIAD based on the pattern of multiple infarcts in a single vascular territory.

## Discussion

The present study compared the imaging features between stroke patients with PFO and those with SIAD. In the full cohort, we found that patients with PFO were older and more likely to have hypertension than patients with SIAD, while the NIHSS score was higher in stroke patients with SIAD than in those with PFO. To minimize bias due to patient selection, we conducted PSM to match patients with PFO and those with SIAD. After PSM, significant difference was not observed in their clinical characteristics but their radiological findings. Single lesions (cortical or subcortical) were more frequently observed in patients with PFO than in those with SIAD, whereas multiple lesions in one vascular territory were more frequently observed in patients with SIAD than in those with PFO. Furthermore, single lesions (corticosubcortical), and posterior circulation involvement were more frequently observed in patients with SIAD than in those with PFO after PSM.

The finding that single lesions (cortical or subcortical) were more frequently observed in the PFO group than in the SIAD group, is consistent to some extent with findings of previous reports (6, 17, 18). Bonati et al. (6) found that among cerebral infarct patients with PFO, 42% (25/60) had single cortical or subcortical infarcts in areas not fulfilling the criteria for territorial infarct, whereas only 6% (2/33) of patients with SIAD had this type of cerebral infarct. This statistical difference is consistent with our findings. In another study (17), it was found that among 25 patients with PFO, 5 (20%) had single subcortical infarct with a diameter over 15 mm, which is significantly different from what was found in the non-PFO group (0%). In the non-PFO group, 8 out of 21 patients showed subcortical infarcts, but none of them showed single subcortical infarcts larger than 15 mm. Kim et al. (18) found that among 117 patients with PFO, 40 had a single subcortical infarct (34.2%), which is significantly different from what was found in patients with atrial fibrillation (11/358, 3.1%). In that study, 46.1% of patients showed large corticosubcortical infarcts, 19% showed confluent large infarcts with additional lesions, and 16.5% showed infarcts in multiple vascular territories. This indicates that atrial fribrillation is more likely to result in infarcts in multiple vascular territories. However, the latter two studies have different baseline information of patients, which leads to bias in their conclusions. In the present study, 50% of patients with PFO had a single cortical or subcortical infarct, which was significantly higher than that of patients with SIAD (21%). The result remained significantly different after propensity analysis (57 vs. 21%). The advantage of the present study was that propensity analysis excluded the bias caused by confounding factors and that the result was more reliable. The underlying mechanism for this finding might be that the patent foramen ovale is relatively small in diameter, and only small thrombi from the periphery can pass it and flow to the carotid or vertebral artery. Therefore, the infarcts are relatively small and often localized to one vascular territory. The other major finding in our study was that multiple lesions in one vascular territory were observed more frequently in the SIAD group than in the PFO group, which was also in line with Bonati's study which showed that territorial infarcts (with or without additional smaller lesions in the same territory) were more likely to occur in patients with arterial dissection (61%, 20/33 vs. 27%,16/60). It is likely that carotid artery dissection or vertebral artery dissection often leads to artery to artery embolism, which subsequently results in multiple infarcts in one vascular territory. A large observational study showed that there was no association between PFO and multiple acute strokes in patients with PFO and cryptogenic strokes (19). Our results are consistent with their findings in that PFO predominantly leads to single infarcts. Calculating the area under ROC curve might be a good way in predicting the likelihood of suffering from PFO or SIAD. Based on the discussion above, we could conclude that single lesions (cortical or subcortical) could be a specific feature of stroke patients with PFO, and multiple lesions in one vascular territory a specific feature of stroke patients with dissection, which could provide further support for differentiating diagnosis of the two etiologies.

After dividing patients with SIAD into carotid dissection and vertebral dissection, it was found that infarcts were more frequently observed in the posterior circulation of SIAD patients than in PFO patients, which was consistent with previously published results (20). However, patients with PFO were more likely to have both anterior and posterior circulations involved than SIAD patients with only one patient in our SIAD group having both circulations involved. The possible reason for this was that PFO tends to result in paradoxical cardioembolic strokes. Emboli from the heart might block both anterior and posterior circulations simultaneously, while emboli from artery dissection only block anterior circulations or posterior circulation, although no statistical significance was observed in our study due to the size limit. Therefore, large-size studies are needed to show whether patients with PFO have more anterior circulation or posterior circulation involved than patients with SIAD.

The present study has several limitations. Firstly, it was a single-center study and the sample size was relatively small. After PSM, the number of patients was reduced to 21 in each group. Thus, a large-scale multi-center study is essential to confirm our conclusion. Secondly, a possible bias might arise from patient selection for TEE, because TEE was an invasive procedure, some suspicious mild PFO associated stroke might be excluded. Thirdly, the cost of MRI scan is high, as a result, some small hospitals cannot perform this test for patients. Fourthly, a study published by New England Journal of Medicine showed that 30 days ambulatory ECG monitoring improves the diagnosis of atrial fibrillation (21). The present study only monitored the patients for 24 h, and the positive diagnosis of atrial fibrillation is low, which might lead to bias in our analysis.

In conclusion, single lesions (cortical or subcortical) might be a typical feature of PFO associated strokes, while multiple lesions in one vascular territory might be a specific feature of arterial dissection associated strokes.

## Ethics Statement

This study was carried out in accordance with the recommendations of the Ethics Committees of the First Affiliated Hospital of Wenzhou Medical University with written informed consent from all subjects. All subjects gave written informed consent in accordance with the Declaration of Helsinki. The protocol was approved by the Ethics Committees of the First Affiliated Hospital of Wenzhou Medical University.

## Author Contributions

YB designed the study. YH and XZ collected the data. JZ and BS analyzed the data. YH and YC drafted the manuscript. HL analyzed the data and edited the manuscript. YB and YH approved the final version of the manuscript. All authors have read and approved the final manuscript.

### Conflict of Interest Statement

The authors declare that the research was conducted in the absence of any commercial or financial relationships that could be construed as a potential conflict of interest.
